# Empyema.—Report of Cases

**Published:** 1910-04

**Authors:** W. D. Gaines

**Affiliations:** La Fayette, Ala.


### EMPYEMA—REPORT OF CASES.

BY W. D. GAINES, M. D., LA FAYETTE, ALA.

This condition is characterized by the presence of pus in the pleural cavity. Purulent pleurisy may occur primarily as a result of a pyogenic infection of the pleura through an open wound or by means of infection conveyed to that membrane through the blood-current.. It also occurs secondarily from infection reaching the pleura from a neighboring focus, as a pneumonia or abscess of the lung, modiastinal disease or from an infected focus in the chest wall. With the exception of Empyema due to a penetrating wound, which may be purulent from the beginning, most cases of Empyema begin with an exudate serum in the pleural cavity, which at a later period becomes purulent from an increase in the number of leukocytes.

The symptoms of Empyema are, in the main, those of septic toxemia plus those of a pleural effusion and a compressed lung.

In the beginning of most cases we have, there are symptoms of pleurisy or pneumonia. Sooner or later there will be evidences of embarrassed respiration associated with the characteristic signs of pus. I will not emphasize the symptoms of this disease in this short paper, but will insist on an early diagnosis of Empyema, which is usually easy, in your cases of "delayed" pneumonia. When you fail to get resolution after the usual time, fever continues for days and even weeks, respirations rapid, may or may not have chills, but in all, your patient is not getting well, examine carefully til you find pus in the pleural cavity. The pus produced in these cases is generally thick and creamy; that produced by the streptococcus is thin and watery. In tuberculous Empyema the pus is also thin and watery but contains masses of caseous material. In all cases of Empyema the pleura is thickened and is covered by an extensive fibrinous exudate.

The prognosis of Empyema depends upon several factors, but in the main should be good. In most children as well as adults the disease is generally the result of pneumococus infection and is not accompanied by a high degree of toxemia and if recognized and treated early the outlook is favorable. Streptococcus and staphylococcus infections of the pleaura, on the other hand, give rise to more virulent toxemia and are more apt to be followed by a general blood infection. In these cases the prognosis is not so favorable.

The treatment of this disease we wish to emphasize and say is purely surgical. We wish also to say that in all surgery no subject has made more advances. Just think, that even three decades ago surgery of the thorax, particularly the pleural cavity, was looked upon as a dangerous field of operation. And it has not been long since the entrance and exit wounds of the chest were sealed as quickly as possible to prevent the entrance of air. What has made all this stride in thoracic surgery? The same modern aseptic surgery that has made it true of all other fields.

Pus in the pleural cavity, as elsewhere, should be drained out as quickly as can be done after a diagnosis has been made. The earlier this is done after its formation, the less adhesions and the better expansion of the lung after its removal, and this one point I wish to remind you is very important. The operation for this disease should be of the most perfect technic. Make your opening in the axilary line, or preferably two or three inches posterior to this line, about the eighth or ninth rib, or always nearest to the bottom of the pus sac. Make a free opening, always resecting one or two ribs, and do not content yourself at any time with a small opening. After the cavity has been well drained, then introduce a large rubber drainage tube and fastein in situ. Change dressing each day, and after several days wash out cavity with a sterile solution of boric acid, and shortening the drain tube each day to allow expansion of the lung and to prevent sinuses and chronic abscess. Now this is another point I wish to emphasize. I believe a large percentage of the cases with chronic discharge is due to the failure to gradually draw the drainage tube. In all of my cases this way recovery has been compelte.

*Case No.* 1.—Willie C., age 12, sick with pneumonia three weeks, one rib resected, drained small quantity two weeks. Recovery perfect.

*Case No.* 2.—J. T. T., age 25, sick with grip-pneumonia five weeks. Then presenting all symptoms of pleural effusion, one rib was resected, letting out about one and one-half pints of pus, drained six weeks with perfect recovery.

*Case No.* 3.—C. L., age 7, sick with pneumonia three weeks, symptoms of pleural effusion present, one rib was resected and about one quart of pus drained out. Drainage continued about eight weeks and made recovery.

*Case No.* 4, L. W. T., age 36, was taken sick with a violent pain in left side followed by high fever, both of which continued without much relief under medical treatment and without any other symptoms of pneumonia present for more than two weeks, when a diagnosis of pus in the pleural cavity was made. Operation was at once performed· more than a pint of pus removed, drainage continued for about six weeks ending in complete recovery.

In cases in which these sinuses or fistulae persist, however in which formerly the method of Estlander gave the most benefit, I would now advise the treatment recently introduced by Dr. Emil Beck.

This method consist in filling the sinus or pus cavity with one of two solutions of bismuth subnitrate and keeping this in position by plugging the outer opening with gauze.

Solution No. 1 consists of one part of arsenic-free subnitrate of bismuth and two parts of sterile amber vaseline.

Solution No. 2 contains sixty parts of amber vaseline and ten parts of parrafine of sufficient hardness to give the mass a fair degree of firmness at the body temperature.

No. 1 is injected every second day until suppuration has almost disappeared, then No. 2 is injected in its place. The injections are repeated as often as is necessary to keep the cavity constantly filled with the mixture. At first it is necessary to do this every day or every second day, then every third and so on until it may be necessary to inject not oftener than once a week or ten days.

*Discussion on paper of Dr. W. D. Gaines, on Empyema.*

Dr. Hugh M. Lokey, Atlanta, Ga., led the discussion.—As to bismuth paste as an injection in Empyema; do not know what effect it will have in the pleural cavities, but have seen it used in tubercular cavities. Have also seen in used in surgical work with very beautiful results. It seems that this bismuth paste gives supporting power to tissue surrounding tubercular cavities. It is perfectly harmless, even in the largest quantities; no irritation. It is simply used, requires a strong heavy metal syringe to force it in; a preparation anyone who can use scales can measure out the ingredients and use himself.

Dr. H. S. Bruce, Opelika, Ala.—There is no question about the treatment of Empyema. In the early stages perfect drainage is necessary, and the character of drainage depends somewhat upon the infection. Our incision should be free, even more so than in other cases. I treat them as I do cases of Tubercular Peritonitis, possibly with as good results as we get from exploratory operations of abdominal Tuberculosis. Wherever we find Tubercular Empyema, our work should be based upon the same principles. Where a large percentage of these cases are tubercular, we make no mistake in making free incision. Have had no experience with bismuth.

Dr. A. L. Harlan, Alexander City, Ala.—I am just a medical man, but have operated on several cases of Empyema. My idea in discussing this subject was to have the surgical side, but it, like all other surgical cases, the medical man is first on the ground, therefore much of the importance rests with us after all. The cases of which I speak were not tubercular, but followed pneumonia and LaGrippe. One case, a babe less than a year old, very ill from the beginning, badly toxic. I made a nice operation I thought, drained it thoroughly, but the babe went on weakening, could not nourish it, so it died.

An early diagnosis is necessary, and we should hunt for Empyema in every case of pneumonia that does not recover rapidly. When hurried respiration is heard, temperature up, and the general symptoms hold on after the eighth day, I begin to suspect Empyema. Main symptoms are dullness on percussion, hacking cough, etc. I was called in consultation with physician ten miles away, who had a patient sick with pneumonia three weeks, and I told him she had Empyema, but I could not find enough flatness. Dr. Goggans and I worked one or more hours, finally localized the place, and we cut right over that place, put in a double drainage tube full of holes, took this out every other day and dressed it. Resected ribs very succesfully. In operating this way, make the opening near the bottom of the cavity.

Dr. Jas. S. McLester, Birmingham, Ala.—I agree with Dr. Harlan that it is the medical man who usually sees the patient first, and with him rests largely the diagnosis. We have so many pneumonias during the winter and the majority of cases of Empyema follows pneumonia that has hung on after two weeks or more.

Dr. W. L. Cooke, Columbus, Ga.—This subject of Empyema is certainly an important one. In my experience suppose I have had ten or twelve cases, the usual method of treatment in all cases is incision and drainage, but it takes six weeks or three months for healing. I wish some means for healing up the sinuses more rapidly in these cases could be gotten hold of. As to the injection of bismuth and vaseline, I have never tried it.

Dr. J. P. Watkins, Opelika, Ala.—Formerly we considered all pleurisies as of tubercular origin. The Empyemas following grippe or pneumococcus infection, can be cured either by aspiration or operation, frequently with no fatalities. The Empyema due to streptococcus requires operation, with frequent fatalities.

In reference to bismuth paste, I tried it on one patient, and got a fine case of poison. These sinuses, if not too old, can be cured by being repeatedly filled with balsam and castor oil, 90 and 10.

Dr. W. D. Gaines, in closing:

I have nothing special to say, but want to thank the gentlemen for discussing the paper, as I get more out of the discussions than anything. I think the double tube a good idea, but have always found one tube satisfactory. To Dr. Watkins I wish to say that Dr. Beck suggests where you have poisoning, inject hot olive oil, and remove at once. Fill cavities with same.

